# Pure Drinking Water

**Published:** 1899-11

**Authors:** 


### Pure Drinking Water.

Thams describes the condition as to drinking water in North Dakota, and points out the very common methods of contamination, both in towns and rural districts. He also gives the results of the analysis of some of the water from the surface wells and artesian borings, showing the proportion of solids contained. Both are overloaded with mineral salts, and he finds, as a result of his routine examination, that the urine of his office patients averages high in specific gravity and is very often alkaline or neutral in reaction. This means, he thinks, that the kidneys of all these patients are overtaxed, and he lays this to the high percentage of solids in the water drank. To verify his findings he has obtained the results of life insurance examinations of one of his colleagues and finds them to agree very closely with his own. In order to furnish pure drinking water free from these excesses of salts, he has devised tablets which may be added to distilled water to give it the proper proportions and make it a safe and healthy drink. These tablets contain each $\frac{1}{4}$ grain sodium sulphate, $\frac{1}{2}$ grain sodium chlorate, $\frac{3}{4}$ grain sodium carbonate, 1 grain magnesium carbonate, and $3\frac{1}{4}$ grains calcium bicarbonate. The alternative of water thus prepared will be found in rainwater from properly constructed cisterns.—*Northwestern Lancet.*

